# From Functional Mapping to Functional Recovery: The Emerging Role of Neuronavigated rTMS in Neurorehabilitation

**DOI:** 10.3390/brainsci16070721

**Published:** 2026-07-06

**Authors:** Marcin Karol Setlak, Bartłomiej Błaszczyk, Krzysztof Suszyński, Sylwia Szostek-Rogula, Maciej Wojtacha, Adam Rudnik

**Affiliations:** 1 Department of Neurosurgery, University Clinical Center, Faculty of Medical Sciences in Katowice, Medical University of Silesia, 40-752 Katowice, Poland; 2 Department of Sports Medicine and Physiology of Physical Exercise, Faculty of Health Sciences in Katowice, Medical University of Silesia, 40-752 Katowice, Poland; 3 Department of Neurological Rehabilitation, University Clinical Center in Katowice, 40-752 Katowice, Poland

**Keywords:** navigated transcranial magnetic stimulation, repetitive transcranial magnetic stimulation, neurorehabilitation, stroke, brain tumors, neuroplasticity

## Abstract

**Highlights:**

**What are the main findings?**

- Neuronavigated rTMS may improve the precision, reproducibility, and documentation of stimulation targets in neurorehabilitation.
- Current clinical evidence remains strongest for conventional rTMS protocols, particularly in post-stroke motor recovery, whereas data on neuronavigated protocols are still limited.

**What are the implications of the main findings?**

- Neuronavigation should be viewed as a precision-enhancing component of rTMS-based rehabilitation rather than as an independent therapeutic breakthrough.
- Postoperative neuro-oncological rehabilitation represents a clinically relevant but still investigational setting for individualized neuronavigated rTMS workflows.

**Abstract:**

**Background/Objectives:** Repetitive transcranial magnetic stimulation (rTMS) has been increasingly investigated as an adjunctive intervention in neurorehabilitation, particularly for motor recovery after stroke. However, conventional rTMS protocols remain limited by variability in target localization, inter-individual anatomical differences, lesion-related network reorganization, and limited reproducibility across treatment sessions. Neuronavigated repetitive transcranial magnetic stimulation (nrTMS) integrates structural neuroimaging with real-time coil tracking, enabling more precise and reproducible stimulation of patient-specific cortical targets. This approach may be especially relevant in patients with focal brain lesions, postoperative anatomical distortion, or functionally reorganized networks. **Methods:** This narrative review summarizes the biological rationale, current clinical evidence, practical workflow, and limitations of nrTMS in neurorehabilitation, with particular attention to the distinction between conventional rTMS and neuronavigated protocols. **Results:** The strongest evidence for rTMS-based rehabilitation remains in post-stroke motor recovery, although most studies have used non-navigated protocols. In contrast, postoperative neuro-oncological rehabilitation represents a clinically relevant but still investigational context for nrTMS, as preoperative functional mapping, postoperative deficits, and early rehabilitation can be integrated within a patient-specific therapeutic pathway. Early studies suggest feasibility when stimulation is combined with structured physiotherapy; however, the available evidence is based on small and heterogeneous cohorts, and clinically meaningful superiority over conventional rTMS or standard rehabilitation has not yet been established. Data in traumatic brain injury, multiple sclerosis, ataxias, and neurodegenerative disorders are still preliminary and heterogeneous. **Conclusions:** Neuronavigation should not be interpreted as an independent therapeutic breakthrough, but rather as a precision-enhancing component of rTMS-based rehabilitation. Its main potential value lies in improving targeting accuracy, session-to-session reproducibility, and integration with individualized neuroimaging and rehabilitation goals. Accordingly, nrTMS should currently be considered a precision-enhancing and hypothesis-generating framework rather than an established rehabilitation standard.

## 1. Introduction

Repetitive transcranial magnetic stimulation (rTMS) is one of the most extensively investigated non-invasive brain stimulation techniques in neurological rehabilitation. Its rationale is based on the ability to modulate cortical excitability and thereby influence neural networks involved in motor and cognitive recovery after central nervous system injury [1]. Among rehabilitation indications, post-stroke motor recovery has received the greatest attention. Nevertheless, the magnitude of benefit, optimal timing, stimulation target, and criteria for patient selection remain incompletely defined. Current evidence-based recommendations support the use of rTMS in selected conditions, including post-acute motor stroke, while also emphasizing that the level of evidence varies considerably across indications and stimulation protocols [2,3].

In both clinical and research settings, rTMS is still frequently delivered using scalp-based landmarks, motor hotspots, or standardized coordinate systems [4,5]. These methods are practical and widely available, but they provide only an indirect approximation of the intended cortical target. This limitation becomes particularly relevant in neurorehabilitation, where stimulation is often applied to networks affected by focal lesions, surgical intervention, compensatory reorganization, or postoperative anatomical distortion. In such patients, variability in treatment response may reflect not only biological heterogeneity, but also uncertainty about whether the same functionally relevant cortical region is being stimulated consistently across sessions [6,7].

Neuronavigated transcranial magnetic stimulation integrates individual neuroimaging with real-time coil tracking, allowing stimulation to be planned, delivered, and documented in relation to the patient’s own cortical anatomy [8]. Neuronavigation does not change the biological mechanism of rTMS itself, but it may reduce one clinically important source of variability: inaccurate or poorly reproducible target localization. This distinction is essential, because most neurorehabilitation studies have evaluated conventional rTMS rather than neuronavigated rTMS specifically. For this reason, neuronavigation should currently be understood not as a separate therapeutic intervention, but as a precision-enhancing component of rTMS-based rehabilitation. Methodological studies support its ability to improve targeting accuracy and precision, although improved technical targeting should not be assumed to automatically translate into superior clinical outcomes [6].

Despite increasing interest in precision neuromodulation, several clinically important gaps remain insufficiently addressed. First, there is no standardized rehabilitation-oriented workflow defining how nrTMS targets should be selected, documented, and integrated with physiotherapy, occupational therapy, or speech and language therapy [1,3]. Second, patient selection criteria remain poorly defined, particularly in individuals with focal lesions, postoperative anatomical distortion, or network reorganization [6]. Third, the literature often discusses improved targeting accuracy and potential clinical benefit together, although these are not equivalent outcomes [6]. Therefore, a clinically oriented synthesis is needed to clarify what neuronavigation currently adds to rTMS-based rehabilitation, where evidence is available, and where assumptions remain largely conceptual or hypothesis-generating.

The potential value of neuronavigation may be greatest in patients whose anatomy and functional organization are no longer reliably represented by standard scalp landmarks. This includes patients with focal brain lesions, lesion-related cortical reorganization, or postoperative anatomical changes. In this context, postoperative neuro-oncological rehabilitation provides a particularly relevant model. In patients undergoing surgery for tumors in eloquent areas, preoperative nTMS is already used for functional mapping and surgical planning. The same patient-specific mapping logic may then be extended into the postoperative period, where navigated repetitive stimulation has been investigated as an adjunct to early rehabilitation in patients with new or worsened motor deficits after glioma or supratentorial tumor surgery [9,10,11]. Early randomized and sham-controlled studies suggest that this approach is feasible and may be associated with motor improvement in selected patients, although the available evidence remains limited and should not yet be generalized beyond specialized clinical settings [9,10].

This narrative review summarizes the biological rationale, current evidence, practical workflow, and limitations of neuronavigated rTMS in neurorehabilitation. The aim is not to present nrTMS as a proven stand-alone therapeutic intervention, but to critically define its emerging role as a precision-enhancing component of rehabilitation-oriented neuromodulation. We focus on clinical areas in which rTMS-based rehabilitation has been studied most extensively, while distinguishing evidence for conventional rTMS from the more limited evidence for neuronavigated protocols. Particular attention is given to stroke rehabilitation and postoperative neuro-oncological rehabilitation, because these fields provide the most relevant clinical framework for discussing whether precision targeting may help translate functional mapping into functional recovery.

## 2. Materials and Methods

This article was designed as a narrative review. A structured but non-systematic literature search was performed to identify relevant publications concerning the use of repetitive transcranial magnetic stimulation (rTMS), navigated transcranial magnetic stimulation (nTMS), and neuronavigated repetitive transcranial magnetic stimulation (nrTMS) in neurological rehabilitation. The search was conducted in PubMed/MEDLINE (National Library of Medicine, Bethesda, MD, USA), Scopus (Elsevier, Amsterdam, The Netherlands), and Google Scholar (Google LLC, Mountain View, CA, USA), and was last updated in May 2026. Only articles published in English were considered. Additional references were identified manually from the reference lists of relevant reviews, clinical studies, and evidence-based guidelines.

The initial database search identified approximately 180 potentially relevant records. After removal of duplicates and clearly irrelevant records, 82 publications were screened at the title and abstract level. Full texts were assessed when the publication addressed rTMS, nTMS, or neuronavigated stimulation in relation to neurorehabilitation, functional recovery, stimulation targeting, or postoperative neurological deficits. A total of 39 publications were included in the narrative synthesis, including clinical trials, systematic reviews, meta-analyses, evidence-based guidelines, methodological studies on neuronavigation and targeting accuracy, and selected studies relevant to postoperative neuro-oncological rehabilitation. The following search terms were used alone and in combination: “repetitive transcranial magnetic stimulation”, “rTMS”, “navigated transcranial magnetic stimulation”, “nTMS”, “neuronavigated rTMS”, “nrTMS”, “neurorehabilitation”, “stroke rehabilitation”, “motor recovery”, “functional recovery”, “brain tumor”, “glioma”, “postoperative rehabilitation”, “traumatic brain injury”, “multiple sclerosis”, “Parkinson’s disease”, “ataxia”, “neuroplasticity”, “neuronavigation”, and “functional mapping”. Priority was given to randomized controlled trials, systematic reviews, meta-analyses, evidence-based guidelines, and methodological studies addressing stimulation targeting, neuronavigation, functional mapping, and rehabilitation outcomes.

Because the aim of this review was to provide a clinically oriented overview rather than a quantitative synthesis, no formal meta-analysis or systematic risk-of-bias assessment was performed. Particular attention was paid to distinguishing evidence obtained with conventional rTMS from evidence specifically involving navigated or neuronavigated protocols. This distinction was considered important because most rehabilitation studies have evaluated conventional rTMS, whereas clinical evidence for nrTMS remains more limited and indication-specific.

## 3. Biological and Clinical Rationale for rTMS-Based Neurorehabilitation

Repetitive transcranial magnetic stimulation is not intended to replace conventional neurorehabilitation. Its potential role is better understood as an adjunctive intervention that may modify cortical excitability and make selected neural networks more responsive to training [1,12]. This distinction is clinically important. Functional recovery after stroke, tumor surgery, traumatic brain injury, or other central nervous system disorders depends mainly on repeated, task-oriented rehabilitation, but the effect of such training may be influenced by the excitability state of the underlying cortical and subcortical networks [13]. In this context, rTMS is used not as an isolated treatment, but as a possible priming tool that may facilitate rehabilitation-induced plasticity when applied together with physiotherapy, occupational therapy, or speech and language therapy.

The physiological effects of rTMS depend on several interacting factors, including stimulation frequency, stimulation pattern, intensity, cortical target, coil orientation, and the baseline excitability state of the stimulated network [1,14]. In clinical practice, low-frequency stimulation is commonly applied with the intention of reducing cortical excitability, whereas high-frequency stimulation and intermittent theta-burst stimulation are often used with the intention of increasing excitability [1,14]. However, this frequency-based distinction should be interpreted as a practical convention rather than a fixed biological rule. Both low- and high-frequency protocols induce neuronal activation during stimulation, and the net after-effects depend on the recruited neuronal populations, inhibitory and excitatory interneuronal circuits, stimulation dose, ongoing network state, and behavioral context. Consequently, changes in motor-evoked potential amplitude or clinical response may vary between individuals and cannot be inferred from stimulation frequency alone. In neurorehabilitation, these effects are commonly interpreted through the concept of activity-dependent plasticity: stimulation may transiently shift the excitability of selected networks, creating a time-limited window in which task-oriented therapy can be more effective [12,15]. One of the most commonly discussed models in rTMS-based neurorehabilitation is the concept of interhemispheric imbalance after unilateral brain injury [15,16]. According to this model, reduced excitability of the lesioned hemisphere may be accompanied by excessive inhibitory influence from the contralesional hemisphere, which can interfere with functional recovery. This concept has supported two main therapeutic strategies: inhibition of the contralesional hemisphere with low-frequency stimulation, or facilitation of the lesioned hemisphere with high-frequency stimulation or intermittent theta-burst stimulation [1,12]. Although this model has been influential, it should be regarded as a useful clinical framework rather than a universal explanation of post-injury recovery. The optimal stimulation strategy may depend on lesion location, corticospinal tract integrity, time from injury, baseline motor output, and the degree of spontaneous or therapy-induced network reorganization [13]. These terms refer to the intended direction of the net physiological after-effect rather than to a simple absence or presence of neuronal activation during stimulation.

From a rehabilitation perspective, the timing of stimulation in relation to training is particularly important [12,15]. rTMS may transiently alter cortical excitability, but functional recovery requires repeated behavioral practice capable of shaping motor, language, or cognitive networks. For this reason, many protocols apply stimulation immediately before or in close temporal proximity to physiotherapy, occupational therapy, or speech and language therapy [1]. This approach is based on the assumption that rTMS may prepare the stimulated network for subsequent training, rather than produce meaningful functional improvement on its own. In practical terms, the therapeutic value of rTMS-based neurorehabilitation should therefore be assessed not only by stimulation parameters, but also by the quality, intensity, timing, and functional relevance of the accompanying rehabilitation program [12,13].

## 4. What Neuronavigation Adds to Conventional rTMS

Conventional rTMS protocols often rely on scalp landmarks, motor hotspots, or standardized coordinate systems to define the stimulation target [4,5]. These approaches are practical and remain widely used, but they provide only an indirect approximation of the underlying cortical anatomy and functional organization. In healthy subjects, this may be acceptable for some research or clinical purposes, but in neurorehabilitation the situation is often more complex. Lesions, postoperative changes, cortical atrophy, and functional reorganization may alter the relationship between scalp landmarks and the cortical regions intended for stimulation [6]. As a result, two patients treated according to the same nominal protocol may in fact receive stimulation over different anatomical or functional targets.

Neuronavigation addresses this problem by linking stimulation to individual structural imaging and by tracking coil position in real time [6,8]. In practice, this allows the operator to define a cortical target on the patient’s own MRI, monitor coil position and angulation during stimulation, and reproduce the same target across repeated sessions [4,5]. This is particularly relevant when rTMS is used as part of a rehabilitation program, where treatment usually requires multiple sessions and where small differences in coil placement may accumulate over time. Neuronavigation also makes the stimulation procedure more transparent: the stimulated region can be documented, reviewed, and, when clinically justified, adjusted according to treatment response or additional imaging data [6].

A further distinction is required between navigational accuracy and stimulation focality. Neuronavigation can improve the spatial accuracy and reproducibility with which the coil is positioned over a predefined cortical target, but it does not by itself determine the spatial extent of the induced electric field [6,17,18]. Stimulation focality depends on additional factors, including coil geometry, coil-to-cortex distance, individual cortical anatomy, tissue conductivity, stimulation intensity, and coil orientation [17,18]. For example, figure-of-eight coils provide relatively focal stimulation compared with less focal coil designs, but even with accurate neuronavigation the induced electric field may spread beyond the visually selected cortical point [17,18]. This distinction is particularly important in patients with cortical atrophy, postoperative anatomical changes, lesions, or altered brain morphology, in whom the relationship between scalp position, cortical target, and effective electric-field distribution may be less predictable.

It is therefore important not to overstate the current evidence. Better target localization is a methodological advantage, but it should not be equated with more focal stimulation or superior functional outcomes. Whether neuronavigation improves the clinical efficacy of rTMS-based neurorehabilitation depends on several factors, including the biological relevance of the selected target, the stimulation protocol, the induced electric-field distribution, the timing of treatment, and the type and intensity of the accompanying rehabilitation. At present, the added value of neuronavigation is best supported as an improvement in targeting precision, reproducibility, and documentation, whereas its superiority over conventional rTMS in terms of functional recovery still requires further controlled studies [1,6].

## 5. Clinical Applications in Neurorehabilitation

### 5.1. Stroke Rehabilitation

Stroke remains the most extensively studied neurological indication for rTMS-based rehabilitation [1,2]. Most clinical studies have focused on motor recovery, especially upper-limb function, although stimulation has also been investigated in post-stroke aphasia, neglect, dysphagia, and cognitive impairment [1]. The rationale for using rTMS after stroke is based on the possibility of modulating excitability within motor and language networks during a period of spontaneous and therapy-induced plasticity. However, the available evidence should be interpreted with caution. Studies differ substantially in terms of stroke phase, lesion location, stimulation target, frequency, number of sessions, outcome measures, and the intensity of concomitant rehabilitation [2,3].

Two broad stimulation strategies have most commonly been used in post-stroke motor rehabilitation. The first aims to shift the excitability balance by applying low-frequency stimulation, usually around 1 Hz, to the contralesional hemisphere. The second aims to enhance excitability within the lesioned hemisphere or perilesional motor network using high-frequency stimulation or intermittent theta-burst stimulation [1,12,15]. Both approaches are based on the interhemispheric imbalance model, but neither should be applied mechanically to all patients [13]. In patients with extensive corticospinal tract damage, severe cortical destruction, or poor residual motor output, increasing excitability of the lesioned hemisphere may not be sufficient to improve function. Conversely, suppressing the contralesional hemisphere may be inappropriate in patients who depend on compensatory contralesional activity for residual motor control [13,19].

In the context of stroke rehabilitation, the main potential contribution of neuronavigation is not a different biological mechanism, but more precise and reproducible target localization. This may be relevant when stimulation is directed to perilesional motor regions, premotor cortex, supplementary motor area, or other network nodes that cannot be reliably identified using scalp landmarks alone [6,13]. Neuronavigation may also help document whether repeated sessions are delivered to the same cortical region, which is important when rTMS is combined with structured motor training [4,6]. However, most clinical evidence supporting rTMS after stroke has been obtained with conventional, non-navigated protocols. Therefore, the specific clinical advantage of neuronavigated rTMS over conventional rTMS in stroke rehabilitation remains biologically plausible but not yet firmly established [1,2].

### 5.2. Illustrative Clinical Vignettes from Stroke Rehabilitation Workflows

The following two cases are presented as illustrative clinical vignettes rather than as a case series or evidence of therapeutic efficacy. Their purpose is to demonstrate how neuronavigated stimulation may be incorporated into individualized rehabilitation workflows in patients with different post-stroke deficits. In both cases, stimulation was used as an adjunct to conventional rehabilitation and was not interpreted as an isolated therapeutic intervention.

#### 5.2.1. Vignette 1: Motor Recovery After Right Hemispheric Stroke

A representative 34-year-old male patient was admitted after an ischemic stroke involving the deep structures of the right hemisphere, including the region of the lentiform nucleus, internal and external capsule, and caudate nucleus. On admission, he presented with left-sided hemiparesis, more pronounced in the upper limb, central facial nerve paresis, markedly impaired gait, and an NIHSS score of 7. Large-vessel occlusion was not identified on vascular imaging, and the patient was not eligible for reperfusion therapy because of the completed character of ischemic changes. After treatment in the stroke unit, he was transferred to neurological rehabilitation with residual left-sided paresis, NIHSS 4, and mRS 3.

During inpatient rehabilitation, the patient underwent a three-week neuronavigated stimulation-assisted rehabilitation protocol. The stimulation strategy was based on previously published protocols combining inhibitory stimulation of the contralesional motor cortex with facilitatory stimulation of the ipsilesional motor cortex in post-stroke upper-limb rehabilitation [20,21]. The cortical hand motor representation was identified using neuronavigated motor mapping and motor-evoked potentials recorded from upper-limb muscles. Lower-limb stimulation was not attempted because reliable motor responses were difficult to obtain, consistent with the more medial location of lower-limb motor representations.

The protocol consisted of sequential inhibitory and facilitatory stimulation. Low-frequency stimulation was applied to the contralesional primary motor cortex with the aim of modulating interhemispheric influences toward the affected hemisphere. Facilitatory high-frequency stimulation was then directed toward the ipsilesional hand motor representation related to the stroke-affected network. The protocol was delivered over 15 weekday sessions, corresponding to a three-week inpatient rehabilitation period. Stimulation parameters were selected according to previously reported post-stroke motor rehabilitation protocols and included 1 Hz stimulation for contralesional inhibition and high-frequency stimulation, most commonly 10 Hz, for ipsilesional facilitation. Stimulation intensity was set at 120% of the individual resting motor threshold [20,21]. Each session included 1000 pulses of contralesional inhibitory stimulation and 1000 pulses of ipsilesional facilitatory stimulation. 

During the stimulation-assisted rehabilitation period, the patient performed simple active movements of the paretic upper limb. Each stimulation session was followed by standard inpatient neurorehabilitation, including physiotherapy, task-oriented motor training, occupational therapy and psychological support. At discharge from rehabilitation, improvement was observed in postural stability, gait pattern, and left-sided motor function, with upper-limb strength improving to approximately IV/V on the Lovett scale. This vignette is presented only as a practical example of how neuronavigated stimulation may be temporally and functionally integrated with structured rehabilitation in a real-world clinical workflow, and not as evidence of therapeutic efficacy.

#### 5.2.2. Vignette 2: Language Recovery After Left Hemispheric Stroke

A second illustrative example involved a 59-year-old female patient after ischemic stroke in the territory of the left middle cerebral artery. The initial right-sided motor deficit improved rapidly; however, the patient continued to present with residual language impairment dominated by reduced speech fluency. At the time of referral for stimulation-assisted rehabilitation, she no longer had clinically relevant difficulty with object naming, memory, or sentence construction. Nevertheless, her speech remained effortful and fragmented, which was functionally important because she was professionally active as a teacher and relied heavily on fluent verbal communication in daily work.

Because clinically relevant language impairment was already present, conventional nTMS-based language mapping was limited. Functional MRI was therefore used to support individualized stimulation planning and to identify language-related cortical activation. The stimulation strategy was designed to combine inhibitory stimulation of contralesional right inferior frontal language-homologous regions with facilitatory stimulation of language-related cortical areas in the affected left hemisphere [22,23]. The protocol was followed by speech and language therapy and was adapted to the patient’s dominant functional problem, namely impaired fluency rather than naming failure.

The first stimulation block consisted of low-frequency continuous stimulation of the right inferior frontal gyrus, targeting anterior and posterior regions corresponding approximately to the homologues of Broca-related areas [22,24]. Stimulation was delivered at 1 Hz with an intensity of 110% of the resting motor threshold, with 1000 pulses delivered per session and 10 sessions per cycle. The second block consisted of facilitatory stimulation of the affected left inferior frontal gyrus, again targeting anterior and posterior Broca-related regions [25]. This was delivered using intermittent 20 Hz stimulation at 80% of the resting motor threshold, with 1000 pulses per session and 10 sessions per cycle, using an inter-train interval of 30 s. In the clinical protocol, stimulation was combined with structured speech and language therapy. A subsequent stimulation phase targeting left posterior language-related regions was introduced according to the speech-language therapist’s assessment, with the aim of addressing persistent fluency-related deficits. After a treatment-free interval of approximately three months, the stimulation-assisted rehabilitation protocol was repeated.

**Figure 1:**
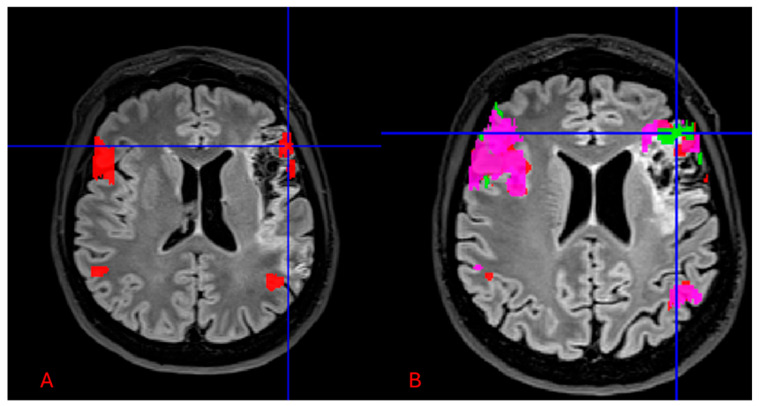
Functional MRI assessment of Broca-related language activation in a 59-year-old patient with residual post-stroke fluency impairment. (**A**) Pre-treatment fMRI obtained during a letter fluency paradigm, showing right-lateralized Broca-related activation with preserved left-hemispheric contribution adjacent to the FLAIR-abnormal region. (**B**) Follow-up fMRI obtained during an antonym-generation paradigm after nrTMS-assisted speech and language therapy, again showing right-lateralized Broca-related activation with residual left-hemispheric contribution. Because different language paradigms and statistical thresholds were used, the figure should be interpreted as an illustrative example of functional imaging-supported rehabilitation planning and follow-up, not as quantitative evidence of treatment efficacy. Colored overlays indicate task-related functional MRI activation maps; crosshair lines are part of the imaging software display and do not represent functional activation.

Follow-up evaluation performed approximately three months after the second treatment cycle included speech-language assessment and functional MRI. No clear clinically significant improvement was documented on formal clinical assessment, although the patient reported subjective improvement in speech fluency and communicative comfort. Functional MRI performed before and after the stimulation-assisted rehabilitation protocol demonstrated Broca-related language activation adjacent to the lateral surface of the FLAIR-abnormal region (Figure 1). In both examinations, the activation pattern was described as right-lateralized, with preserved left-hemispheric contribution, as reflected by lateralization indices of 0.40 and 0.43 for the left hemisphere, respectively. Because different language paradigms and statistical thresholds were used, these findings should not be interpreted as a quantitative pre–post measure of treatment effect. Rather, they illustrate altered post-stroke language network organization and the potential role of functional imaging in planning and monitoring individualized nrTMS-assisted speech rehabilitation.

## 6. Postoperative Neuro-Oncological Rehabilitation

Postoperative neuro-oncological rehabilitation represents a clinically relevant but still investigational setting for discussing neuronavigated rTMS. In contrast to many stroke studies, where stimulation protocols have often relied on conventional targeting methods, patients undergoing surgery for tumors in eloquent brain regions may already have preoperative nTMS maps available as part of functional surgical planning [26,27,28]. These maps can identify motor- or language-related cortical regions and may help define individualized stimulation targets after surgery [26,28]. This creates a practical continuity between preoperative functional mapping, postoperative neurological deficits, and early rehabilitation planning within a patient-specific therapeutic workflow.

The therapeutic rationale for postoperative nrTMS is partly derived from stroke rehabilitation. In patients who develop new or worsened motor deficits after tumor resection, low-frequency stimulation of the contralesional motor cortex has been used to reduce transcallosal inhibition and support recovery of the affected motor network [9,11]. In the available clinical studies, stimulation was typically combined with early physiotherapy rather than used as a stand-alone intervention [9,10,11]. This distinction is important because the goal of postoperative nrTMS is not to replace rehabilitation, but rather to create more favorable conditions for training-induced motor recovery during the early postoperative period.

An additional advantage of this approach is that stimulation targets may be selected not only according to anatomical landmarks, but also on the basis of preoperative functional mapping and postoperative neurological status. In this sense, postoperative nrTMS may be viewed as a potential extension of the functional mapping concept into the rehabilitation phase of patient care. This idea remains preliminary and hypothesis-generating, and should be interpreted as a rationale for further controlled studies rather than as evidence of established clinical efficacy.

Early clinical studies suggest that postoperative nrTMS is feasible in selected brain tumor patients and can be integrated with early physiotherapy [9,10,11]. However, the evidence remains preliminary and should be interpreted cautiously. Available studies include relatively small and heterogeneous cohorts and differ in tumor type, timing of stimulation, severity of paresis, stimulation protocol, rehabilitation intensity, and follow-up duration. Moreover, clinical improvement has also been observed in sham or control groups in some studies, underlining the contribution of spontaneous recovery, early physiotherapy, placebo effects, and study design in this population [10]. Therefore, postoperative nrTMS should currently be regarded as an investigational adjunctive strategy for specialized centers rather than an established standard of care. The following sections summarize exploratory applications of rTMS and neuronavigated rTMS in neurological conditions beyond stroke and postoperative neuro-oncological rehabilitation. These areas are included to illustrate the broader rehabilitation landscape, but the available evidence is generally less mature, more heterogeneous, and less specific to neuronavigated protocols. Therefore, these indications should not be interpreted as established clinical applications of nrTMS-based neurorehabilitation.

## 7. Traumatic Brain Injury

Evidence for rTMS-based rehabilitation after traumatic brain injury remains exploratory and less mature than in stroke. This is partly related to the heterogeneity of traumatic brain injury itself, which may include focal contusions, diffuse axonal injury, cognitive and affective symptoms, motor impairment, fatigue, pain, and disorders of arousal. rTMS has been investigated mainly as a tool for modulating prefrontal, motor, or frontoparietal networks, depending on the dominant clinical problem. In this population, neuronavigation may be useful because structural lesions, diffuse injury, and altered network organization can make standardized scalp-based targeting less reliable. However, available studies remain heterogeneous, and current evidence is insufficient to support the routine use of rTMS or neuronavigated rTMS as a standard rehabilitation intervention after traumatic brain injury [29,30].

## 8. Multiple Sclerosis

Multiple sclerosis is discussed separately because it represents a disseminated inflammatory and neurodegenerative disease model, rather than a single focal lesion model such as stroke or postoperative paresis. In this context, rTMS has been explored mainly as an adjunctive intervention for symptoms such as spasticity, fatigue, gait impairment, and cognitive dysfunction. Available systematic reviews suggest that non-invasive brain stimulation, including rTMS, may have beneficial effects on selected symptoms, particularly spasticity and fatigue, but results remain variable and are based on relatively small and heterogeneous studies [31,32,33]. In this context, neuronavigation may help improve stimulation reproducibility and allow more individualized targeting of preserved cortical or network regions. However, its specific clinical advantage over conventional rTMS in multiple sclerosis has not yet been established [31,32,33].

## 9. Neurodegenerative Disorders and Ataxias

As another group of exploratory applications, rTMS has been investigated in selected neurodegenerative disorders and ataxias, most commonly in Parkinson’s disease and cerebellar syndromes. In Parkinson’s disease, stimulation of the primary motor cortex, supplementary motor area, and other cortical targets has been explored mainly for motor symptoms, although non-motor outcomes have also been investigated [34,35]. In cerebellar ataxias, stimulation has been directed mainly toward cerebellar or cerebello-cortical networks with the aim of modulating motor coordination and balance [36,37]. However, these applications should be regarded as exploratory in the context of neurorehabilitation. The available literature remains heterogeneous with respect to diagnosis, stimulation target, protocol, outcome measures, and treatment duration. Neuronavigation may improve anatomical targeting, particularly for cortical and cerebellar targets, but there is still insufficient evidence that navigated protocols provide superior rehabilitation outcomes compared with conventional rTMS. The main potential clinical applications of rTMS and neuronavigated rTMS in neurorehabilitation are summarized in Table 1.

## 10. Practical Workflow and Implementation in Rehabilitation Settings

**Figure 2:**
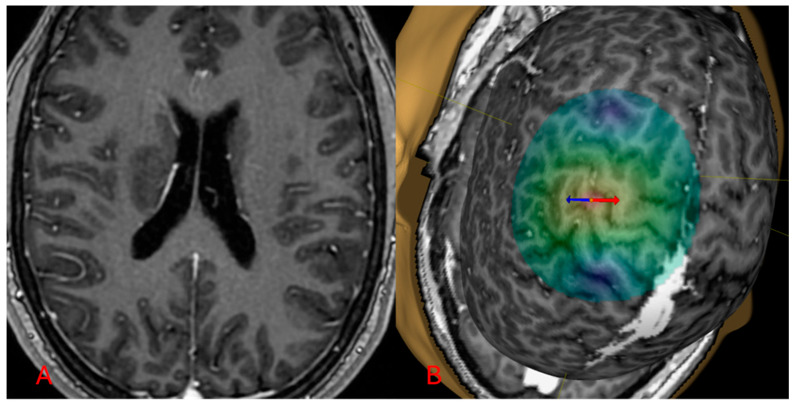
Example of MRI-based and neuronavigated rTMS target selection in post-stroke motor rehabilitation. (**A**) Structural MRI demonstrating focal post-stroke changes. (**B**) Neuronavigated rTMS planning screen showing the contralesional primary motor cortex hand representation selected for low-frequency inhibitory stimulation. This workflow illustrates how lesion imaging and neuronavigated motor mapping may be integrated to define an individualized stimulation target for upper-limb rehabilitation. The colored cortical overlay represents the neuronavigation-based motor mapping/target visualization, and the arrow indicates the selected stimulation direction/target orientation within the planning software.

The clinical implementation of neuronavigated rTMS in neurorehabilitation requires more than the availability of a stimulation device. In practice, it should be organized as a structured workflow that links patient selection, imaging-based target definition, stimulation parameters, and conventional rehabilitation goals. The procedure begins with identification of the dominant functional problem, such as upper-limb paresis, aphasia, neglect, spasticity, gait impairment, or postoperative motor deficit. The stimulation target should then be selected according to the clinical syndrome, lesion anatomy, available neurophysiological data, and the planned rehabilitation intervention. This approach is particularly important because the same anatomical target may not be equally relevant in all patients, even when the clinical deficit appears similar [1,6,13]. An example of MRI-based lesion visualization and neuronavigated selection of a contralesional motor target for inhibitory stimulation is shown in Figure 2.

Once the target has been defined, the stimulation protocol should be planned together with the rehabilitation session rather than independently from it. In most rehabilitation-oriented protocols, rTMS is applied immediately before or in close temporal proximity to physiotherapy, occupational therapy, or speech and language therapy [1,12]. The aim is to use stimulation as a preparatory intervention that may transiently modify cortical excitability and support subsequent task-oriented training [12,13,15]. For this reason, reporting the rehabilitation component is as important as reporting stimulation frequency, intensity, number of pulses, and number of sessions [1,3].

In practical rehabilitation settings, anatomical targeting should always be linked to the functional goal of treatment. For example, stimulation of motor cortical areas may be considered when the main problem is paresis or impaired motor control, whereas prefrontal, inferior frontal, parietal, or cerebellar targets may be relevant for cognitive, language, attentional, or coordination-related deficits. Neuronavigation is useful because it allows these targets to be defined on the patient’s own MRI rather than inferred from external landmarks. Reported stimulation parameters vary considerably, but commonly include low-frequency protocols around 1 Hz, high-frequency protocols between 5 and 20 Hz, or theta-burst stimulation, with intensity usually expressed as a percentage of the individual resting motor threshold [1,6,14].

In neuronavigated protocols, target planning should also consider parameters that influence the effective electric-field distribution rather than only the visually selected cortical point. These include coil type, coil orientation, coil-to-cortex distance, stimulation intensity, and local cortical anatomy [17,18]. When available, electric-field modeling may provide additional information about the likely distribution of stimulation, particularly in patients with postoperative anatomical distortion, cortical atrophy, or lesion-related changes in brain morphology [18]. However, such approaches are not yet standardized for routine neurorehabilitation practice and should currently be regarded as tools for protocol refinement and research rather than mandatory clinical requirements.

Clinical implementation also requires predefined outcome measures and consistent documentation of each stimulation session. Depending on the indication, functional assessment may include upper-limb motor scales, grip strength, gait tests, spasticity scales, language assessment, cognitive screening, or patient-reported measures of fatigue and quality of life. In addition to clinical outcomes, the stimulation protocol should report the target region, method of target identification, coil orientation, stimulation frequency, intensity, number of pulses, number of sessions, timing in relation to rehabilitation, and any adverse effects [1,3]. This level of reporting is particularly important in neuronavigated protocols, because one of the main advantages of neuronavigation is the ability to document and reproduce the stimulated cortical site across sessions [4,6,7].

## 11. Current Limitations and Future Directions

Several limitations should be considered when interpreting the current literature on neuronavigated rTMS in neurorehabilitation. First, most clinical evidence in rehabilitation has been generated using conventional rTMS rather than neuronavigated protocols [1,2,3]. This is particularly important in stroke rehabilitation, where the overall evidence base is larger, but the specific added value of neuronavigation remains insufficiently tested. As a result, conclusions regarding nrTMS are often based on a combination of clinical evidence from conventional rTMS studies and methodological evidence showing improved targeting accuracy with neuronavigation.

Second, available studies are heterogeneous in terms of patient selection, lesion type, time from injury or surgery, stimulation target, frequency, intensity, number of sessions, and outcome measures [1,2,3,10]. This heterogeneity makes it difficult to compare results across studies and to define optimal protocols for specific patient groups. It also limits the possibility of identifying which patients are most likely to benefit from stimulation. Future trials should therefore report not only stimulation parameters, but also lesion characteristics, baseline functional status, corticospinal tract integrity when available, rehabilitation intensity, timing of stimulation in relation to therapy, and the method used for target localization.

Third, the clinical effect of rTMS-based neurorehabilitation is difficult to separate from spontaneous recovery, placebo effects, and the effect of conventional rehabilitation itself [10]. This is especially relevant in the early post-stroke or postoperative period, when neurological improvement may occur independently of stimulation. Sham-controlled designs, adequate follow-up, and clearly defined functional endpoints are therefore essential. In addition, future studies should clarify whether improved anatomical targeting with neuronavigation translates into clinically meaningful functional gains, rather than only better technical precision [6].

Future development of nrTMS-based neurorehabilitation will likely require a shift from purely anatomical targeting toward more functionally and network-informed approaches. Individualized stimulation targets may eventually be refined using combinations of structural MRI, diffusion tensor imaging and tractography, functional MRI, neurophysiological mapping, EEG-based measures of cortical state, and electric-field modeling [18,38]. Such multimodal approaches could help determine not only where stimulation can be delivered most accurately, but also which cortical or network targets are most relevant for recovery in a given patient. In parallel, nrTMS may fit within the broader concept of a Learning Rehabilitation System [39], in which patient-specific functional status, neuroimaging, stimulation parameters, rehabilitation intensity, and longitudinal outcomes are systematically collected and used to refine future therapeutic decisions. At present, however, these strategies remain insufficiently standardized and should be considered directions for future research rather than established clinical practice.

Neuronavigation also has practical and technical limitations that should be explicitly acknowledged. Accurate stimulation depends on the quality of image-to-head registration, stability of head and coil tracking, maintenance of coil orientation, and predefined tolerances for deviations from the planned target [6,18]. During longer rehabilitation sessions, patient movement, fatigue, spasticity, discomfort, or involuntary head motion may reduce positioning accuracy. In addition, tracking systems have finite temporal resolution and may introduce small delays between head or coil movement and visual feedback to the operator. These factors do not invalidate neuronavigation, but they emphasize that navigated stimulation remains operator-dependent and requires systematic quality control, documentation of targeting deviations, and appropriate training.

Finally, implementation of neuronavigated rTMS in routine rehabilitation practice remains limited by practical barriers. These include equipment cost, access to recent structural imaging, time required for target planning and patient registration, operator training, and the need for close cooperation between neurologists, rehabilitation physicians, physiotherapists, speech therapists, neurophysiology teams, and neurosurgical teams. For these reasons, neuronavigated rTMS should currently be viewed as a specialized and still investigational adjunct to neurorehabilitation. Its broader role will depend on standardized protocols, pragmatic clinical trials, and evidence that the additional complexity of neuronavigation provides measurable benefit for patients.

## 12. Conclusions

Neuronavigated rTMS represents a technically refined form of rTMS-based neuromodulation that may be particularly relevant in neurorehabilitation, where anatomical variability, focal lesions, postoperative changes, and functional reorganization can make conventional scalp-based targeting less reliable. Its main current value lies in improving target localization, session-to-session reproducibility, and documentation of the stimulated cortical region. These features may support more individualized rehabilitation protocols, especially when stimulation is combined with structured physiotherapy, occupational therapy, or speech and language therapy.

At present, however, neuronavigation should not be interpreted as proof of superior clinical efficacy. Most rehabilitation evidence still comes from conventional rTMS studies, particularly in post-stroke motor recovery. The most specific emerging clinical framework for nrTMS appears to be postoperative neuro-oncological rehabilitation, where preoperative functional mapping, postoperative deficits, and early rehabilitation can be linked within one patient-specific workflow. Evidence in traumatic brain injury, multiple sclerosis, neurodegenerative disorders, and ataxias remains preliminary and heterogeneous.

Future studies should determine whether the technical advantages of neuronavigation translate into clinically meaningful functional outcomes. This will require well-designed sham-controlled trials, standardized reporting of stimulation and rehabilitation protocols, careful patient selection, and longer follow-up. Until such data are available, nrTMS should be regarded as a specialized and still investigational adjunct to neurorehabilitation rather than an established standard of care. Its future role may depend on whether precision targeting can move beyond improved localization alone and become part of a broader strategy for translating functional mapping into functional recovery.

This translation will require evidence that neuronavigation improves patient-relevant outcomes, not only targeting accuracy, reproducibility, or procedural documentation.

## Figures and Tables

**Table 1:** Potential Clinical Applications of rTMS and Neuronavigated rTMS in Neurorehabilitation.

Clinical Area	Main Rehabilitation Target/Problem	Typical Stimulation Approach	Potential Role of Neuronavigation	Current Evidence/Limitations
Stroke	Upper-limb paresis, aphasia, neglect, dysphagia, cognitive impairment	Low-frequency contralesional stimulation; high-frequency or iTBS ipsilesional stimulation	More precise targeting of perilesional motor regions, premotor cortex, SMA, or functionally relevant network targets	Largest evidence base for rTMS, but mostly non-navigated protocols; added value of neuronavigation not firmly established
Postoperative neuro-oncological rehabilitation	New or worsened motor deficit after tumor resection	Low-frequency stimulation of contralesional M1 combined with early physiotherapy	Use of preoperative nTMS maps; individualized postoperative targeting; integration with surgical workflow	Specific emerging clinical context for nrTMS; early studies suggest feasibility, but evidence remains preliminary and heterogeneous; not standard of care
Traumatic brain injury	Motor, cognitive, affective symptoms, fatigue, pain, disorders of arousal	Prefrontal, motor, or frontoparietal stimulation depending on deficit	May improve target definition in structurally altered or diffuse injury patterns	Exploratory; evidence heterogeneous and less mature than in stroke
Multiple sclerosis	Spasticity, fatigue, gait impairment, cognitive dysfunction	Motor cortex or prefrontal stimulation depending on symptom	Improved reproducibility and individualized targeting of preserved cortical or network regions	Small and heterogeneous studies; specific advantage of neuronavigation unproven
Parkinson’s disease and other neurodegenerative disorders	Motor symptoms, gait dysfunction, non-motor symptoms	M1, SMA, DLPFC, or other cortical targets	More accurate cortical targeting and reproducibility	Exploratory in rehabilitation context; protocols and outcomes heterogeneous
Cerebellar ataxias	Coordination, balance, gait instability	Cerebellar or cerebello-cortical stimulation	More reproducible targeting of cerebellar regions	Preliminary evidence; optimal targets and protocols not established

Abbreviations: rTMS—repetitive transcranial magnetic stimulation; nrTMS—neuronavigated repetitive transcranial magnetic stimulation; iTBS—intermittent theta-burst stimulation; SMA—supplementary motor area; M1—primary motor cortex; DLPFC—dorsolateral prefrontal cortex.

## Data Availability

No new data were created or analyzed in this study. Data sharing is not applicable to this article.

## Abbreviations

The following abbreviations are used in this manuscript:

DLPFC	dorsolateral prefrontal cortex
fMRI	functional magnetic resonance imaging
iTBS	intermittent theta-burst stimulation
M1	primary motor cortex
MEP	motor-evoked potential
MRI	magnetic resonance imaging
mRS	modified Rankin Scale
NIHSS	National Institutes of Health Stroke Scale
nrTMS	neuronavigated repetitive transcranial magnetic stimulation
nTMS	navigated transcranial magnetic stimulation
rTMS	repetitive transcranial magnetic stimulation
SMA	supplementary motor area
TMS	transcranial magnetic stimulation
